# Amplifying mRNA vaccines: potential versatile magicians for oncotherapy

**DOI:** 10.3389/fimmu.2023.1261243

**Published:** 2023-10-23

**Authors:** Chaoying Hu, Jianyang Liu, Feiran Cheng, Yu Bai, Qunying Mao, Miao Xu, Zhenglun Liang

**Affiliations:** ^1^ Division of Hepatitis and Enterovirus Vaccines, National Institutes for Food and Drug Control, Beijing, China; ^2^ National Health Commission (NHC), Key Laboratory of Research on Quality and Standardization of Biotech Products, National Institutes for Food and Drug Control, Beijing, China; ^3^ National Medical Products Administration (NMPA), Key Laboratory for Quality Research and Evaluation of Biological Products, Institute of Biological Products, National Institutes for Food and Drug Control, Beijing, China

**Keywords:** cancer vaccine, amplifying mRNA, tumor-specific antigen, tumor associated antigen, *in vitro* transcription

## Abstract

Cancer vaccines drive the activation and proliferation of tumor-reactive immune cells, thereby eliciting tumor-specific immunity that kills tumor cells. Accordingly, they possess immense potential in cancer treatment. However, such vaccines are also faced with challenges related to their design and considerable differences among individual tumors. The success of messenger RNA (mRNA) vaccines against coronavirus disease 2019 has prompted the application of mRNA vaccine technology platforms to the field of oncotherapy. These platforms include linear, circular, and amplifying mRNA vaccines. In particular, amplifying mRNA vaccines are characterized by high-level and prolonged antigen gene expression at low doses. They can also stimulate specific cellular immunity, making them highly promising in cancer vaccine research. In this review, we summarize the research progress in amplifying mRNA vaccines and provide an outlook of their prospects and future directions in oncotherapy.

## 1 Introduction

Tumors pose a severe threat to human health. Both the suppression and reprogramming of the immune system play key roles in the onset and progression of tumors. Immuno-oncotherapy refers to a series of methods involving the activation of the immune system for cancer treatment. It includes immune checkpoint inhibitors, therapeutic antibodies, adoptive cell transfer, small molecule immunosuppressants, and cancer vaccines (1). Among these methods, cancer vaccines serve as a form of active immunotherapy. Upon vaccination, they activate the specific antitumor immune responses of patients to eradicate tumor cells. In clinical studies, cancer vaccines have demonstrated positive effects in the active immunotherapy of tumors, including the eradication of tumor cells and prevention of tumor metastasis and recurrence (2, 3). However, cancer vaccines, such as peptide vaccines and cell vaccines, have a single antigen, poor antitumor immune activation effect, and high production cost. mRNA vaccines can deliver multiple antigens, induce stronger T cell responses, and have a relatively lower production cost.

In 1995, Conry et al. first proposed the concept of using messenger RNAs (mRNAs) for immuno-oncotherapy (4). The coronavirus disease (COVID-19) mRNA vaccine Comirnaty was approved for marketing by the United States Food and Drug Administration (US FDA) in 2021, making it the first-ever approved mRNA vaccine (5). With the rapid development, manufacturing, and application of mRNA vaccines against COVID-19, increasing attention has been focused on mRNA vaccines over the last few years. Compared with conventional vaccines, mRNA vaccines provide the advantages of rapid editability, low biosafety risk, T-cell activation, and induction of stronger immunogenicity (1, 6) Two mRNA cancer vaccines, mRNA-4157 and BNT122, have recently exhibited good therapeutic effects in clinical studies on melanoma and pancreatic ductal adenocarcinoma, respectively. These findings have led to widespread interest in the use of mRNA vaccines for cancer treatment (7).

In particular, mRNA vaccines include linear mRNA, circular mRNA, and amplifying mRNA vaccines (8). The characteristics of amplifying mRNA vaccines are as follows (1): They are dependent on self-amplifying RNAs (saRNAs) that are capable of synthesizing RNA within host cells using an RNA-dependent RNA polymerase (RdRp). This enables the high-level and prolonged expression of the tumor antigen genes carried by vectors, thereby achieving levels of protein expression similar to those of linear mRNA vaccines but at lower doses and a relatively lower production cost (2); Amplifying mRNAs possess the potential for stimulating immune responses as they can form double-stranded RNAs during the amplification process. This is highly similar to the viral RNA replication process, which can activate the innate immune response of the host and further enhance the effects of the vaccine (3); Amplifying mRNAs can simultaneously express multiple antigens, thereby inducing humoral and cellular immune responses targeted toward different antigens (9, 10). Therefore, amplifying mRNA vaccines offer promising prospects for the research and application of vaccines against infectious diseases and cancer. At present, there are 43 COVID-19 mRNA vaccines in the clinical stage, of which 14 are amplifying mRNA vaccines. Among the various amplifying mRNA-based cancer vaccines, dozens are in the preclinical stage and five are in the clinical stage (11–13).

In this study, we present a review of the research progress and working principles of amplifying mRNA vaccines and highlight the considerations and outstanding issues in the quality control of amplifying mRNA vaccines, so as to provide a reference for the future development of amplifying mRNA cancer vaccines.

## 2 History of amplifying mRNA vaccine research

**Figure 1:**
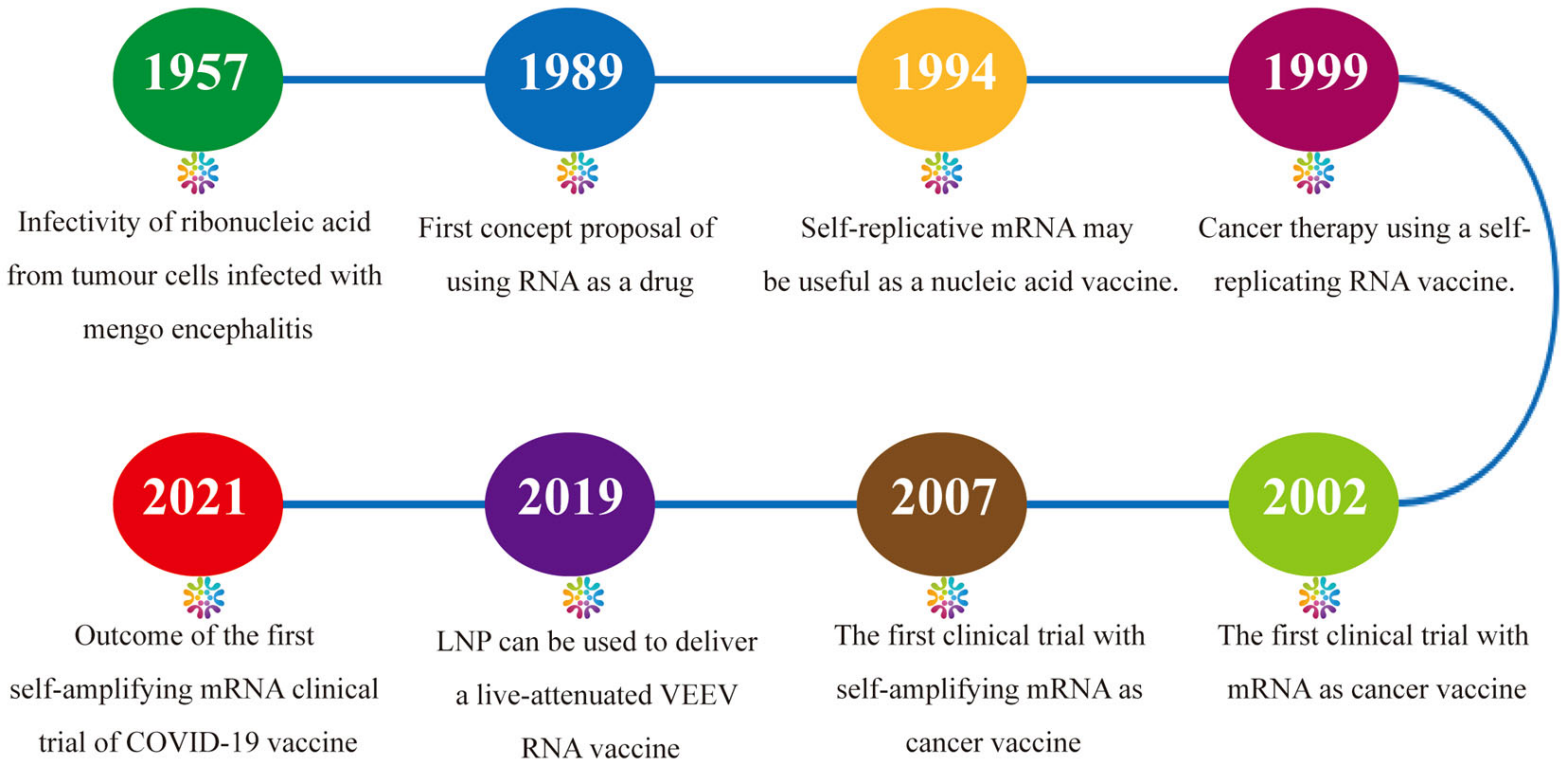
Milestones in the development of amplifying mRNA-based cancer vaccines.

Research on amplifying mRNA vaccines dates back to the investigation of the characteristics of RNA viruses in the 1950s and 1960s. When RNA extracted from ascites tumor cells infected with Mengo encephalitis virus was introduced into cells, it was found that complete infectious viral particles could be synthesized (14). In 1989, Malone et al. conceived the concept of mRNA-based drugs (15). Zhou et al. in 1994 reported the generation of humoral responses with high antibody titers in mice through the expression of the nucleoprotein of influenza virus using the Semliki Forest virus (SFV) replicon, and first proposed the concept of using synthesized amplifying mRNAs as vaccines (16). In 1999, Ying et al. proposed that naked, noninfectious saRNAs may be used for the development of cancer vaccines (17). In 2003, in a phase I clinical trial of an mRNA vaccine encoding prostate-specific antigen (PSA), it was found that the vaccine effectively evoked T-cell-mediated antitumor immune responses *in vivo* (18). The first amplifying mRNA-based therapeutic cancer vaccine AVX701 entered phase I clinical testing in 2007 (19). In 2019, Beissert et al. designed a novel bipartite amplifying mRNA vector system using trans-amplifying RNA (taRNA). Compared with conventional self-amplifying mRNA (samRNA) vaccines, this method has key advantages in the aspects of safety, production, and ease of optimization (20). During the same year, Samsa et al. pioneered the use of lipid nanoparticles (LNPs) for the delivery of a live-attenuated Venezuelan equine encephalitis virus (VEEV) RNA vaccine (21). In 2021, Imperial College London reported the outcomes of a clinical trial of the first amplifying mRNA COVID-19 vaccine worldwide. Among 23 participants who received the vaccine at doses of 5.0 and 10.0 μg, 20 developed immune responses (22). At present, the amplifying mRNA vaccine technology platform has been applied in the clinical research of vaccines for infectious viruses such as influenza, respiratory syncytial virus (RSV), rabies, Ebola, and HIV-1 and for cancers such as melanoma and has exhibited immense potential in the fields of infectious disease prevention and oncotherapy (12, 23) (**Figure 1**).

## 3 Mechanisms of action of amplifying RNA vaccines

Amplifying mRNAs differ from nonamplifying mRNAs in that they can serve as templates for amplification. Besides conventional structural elements such as the cap, 5′-UTR, 3′-UTR, and poly(A) tail, amplifying mRNAs also contain a sequence within the 5′ open reading frame that encodes an RNA-dependent RNA polymerase (RdRp) complex (replicase) and a subgenomic promoter (24). The gene sequences encoding viral structural proteins are usually located downstream of the subgenomic promoter. During the design of amplifying mRNA vaccines, this sequence is replaced by a gene sequence encoding the vaccine antigen of interest (25). The genomes of alphaviruses such as the Venezuelan equine encephalitis virus (VEEV) (26), Sindbis virus (SINV) (27), and Semliki forest virus (SFV) (28) are commonly used in the design of amplifying mRNA vaccines. Other genomes used include those of the tick-borne encephalitis virus (TBEV) (29), yellow fever virus (YFV) (30), and Kunjin virus (KUNV) (31). For instance, the replicase encoded by an alphavirus vector is composed of four nonstructural proteins (NSPs). NSP1 is responsible for the connection between the replicase complex and cell membrane and the 5′-end capping of the viral RNA (32); NSP2 serves as a RNA helicase and protease for polyprotein processing (33, 34); NSP3 mediates various virus-host protein-protein interactions (35); while NSP4 is the RdRp (36). Aggregation of these four NSPs through a complex multistep process causes the formation of an replicase, which provides amplifying mRNA with the ability to self-replicate (37).

**Figure 2:**
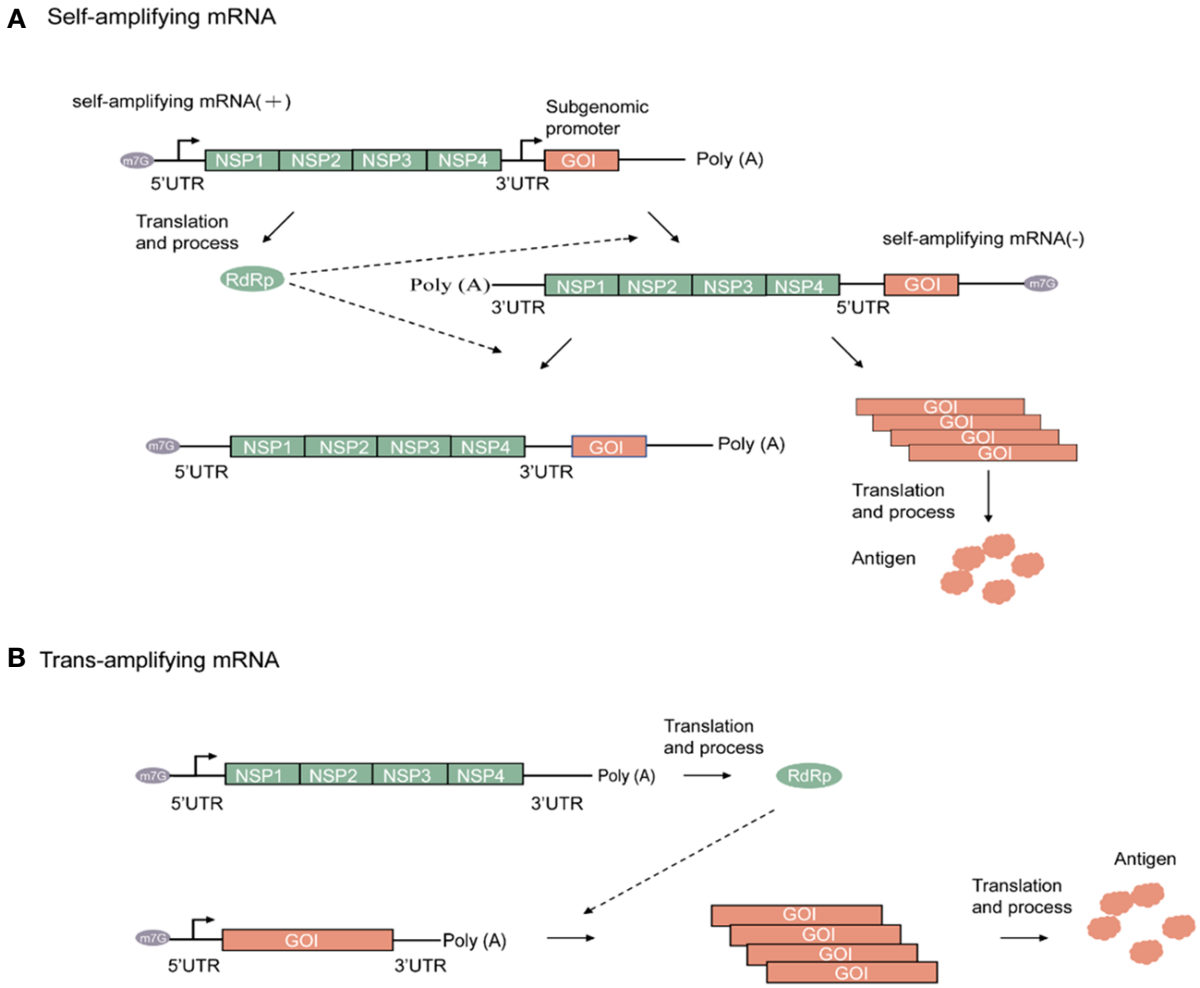
Alphaviruses-based amplifying mRNA expression process. **(A)** Mechanism of self-amplifying mRNA. **(B)** Mechanism of Trans-amplifying mRNA. Green rectangular, Non-structural proteins (NSP) RNA sequence; green oval, RNA-dependent RNA polymerase; orange rectangular, gene of interest RNA sequence; orange cloud, antigen; grey oval, N7-methylguanosine RNA sequence.

Following its entrance in the host cell, the amplifying mRNA is translated in a host ribosome-dependent manner and then undergoes processing within the host cell to form replicase. As the sequence of the amplifying mRNA is a sense (+)-strand RNA, it is used by replicase as a template for amplification, thereby forming the antisense (–)-strand amplifying mRNA. Subsequently, amplification of the (–)-strand amplifying mRNA leads to the synthesis of two different types of (+)-strand RNA. One is a copy of the original full-length genomic RNA, whereas the other is a subgenomic RNA encoding the target gene. Given the presence of a subgenomic promoter and the gene of interest (GOI) in the amplifying mRNA sequence, the viral replicase recognizes the subgenomic promoter in the (–)-strand amplifying mRNA. Consequently, a large amount of (+)-strand mRNA containing the target gene sequence is synthesized, and the required target product is then obtained through translation (23). Hence, amplifying mRNA results in the high-level and prolonged expression of the target protein at low doses. **Figure 2A** shows a schematic diagram of the working principle of a self-amplifying mRNA.

Compared with nonamplifying linear mRNA, amplifying mRNA contains an additional sequence that encodes the replicase. For instance, the replicase sequence in the VEEV-encoded RNA has a length of up to 7 kb (38). As RNA stability is negatively correlated with sequence length, this has resulted in strict requirements for RNA storage and transportation. To address this issue, a split-vector trans-amplifying RNA system can be adopted, that is, the sequence encoding the RdRp complex and that encoding the target gene can be split into two independent transcripts for delivery This contributes to the reduction of mRNA size, thereby enhancing overall mRNA stability (20). The working principle of a trans-amplifying mRNA is shown in **Figure 2B**.

## 4 Progress on amplifying mRNA cancer vaccines

Amplifying mRNAs, which serve as a novel vaccine technology and treatment method, have been increasingly adopted in the fields of infectious disease prevention and oncotherapy. This section summarizes the applications of amplifying mRNA cancer vaccines in preclinical (**Table 1**) and clinical studies (**Table 2**).

**Table 1:** Preclinical studies on amplifying mRNA cancer vaccines.

Source of RdRp	Antigen	Tumor	Method of injection	Animal model	Concomitant drug	Effects	Reference
SFV	E6E7; E6+E7 fusion	HPV-16	Subcutaneous Injection	C57BL/6 mice	/	Tumor regression, complete eradication	(39)
SFV	VEGFR-2	CT26	Subcutaneous Injection	BALB/c mice	IL12; IL-4	Inhibition of tumor growth, metastasis	(40)
SFV	IL18	B16	Intratumoral Injection	C57BL/6 mice	IL12	Enhanced Th1-type response, antitumor immunity	(41)
SFV	IL12	MC38	Intratumoral Injection	C57BL/6 mice	/	Tumor regression in mice	(42)
SFV	IL12	4T1	Intratumoral Injection	BALB/c mice	LVR01	Tumor regression in mice, prolong survival in tumor mice	(43)
VEEV	E7	HPV-16	Subcutaneous Injection	C57BL/6 mice	/	Immune responses, tumor protection in mice	(44)
VEEV	TRP2	Bl6	Subcutaneous Injection	C57BL/6 mice	/	Tumor regression in mice	(45)
VEEV	TRP2	Bl6	Subcutaneous Injection	C57BL/6 mice	GITR mAb; CTLA-4 mAb	Tumor regression in mice	(46)
VEEV	PSMA	TRAMP	Subcutaneous Injection	BALB/c and C57BL/6	/	Robust immune responses in mice	(47)
VEEV	STEAP	TRAMP	Subcutaneous Injection	C57BL/6 mice	/	STEAP-specific immune responses	(48)

SFV, Semliki Forest virus; VEEV, Venezuelan equine encephalitis virus; PMSA, prostate specific membrane antigen; STEAP, six-transmembrane epithelial antigen of the prostate; TRAMP, transgenic adenocarcinoma mouse prostate; VEGFR-2, vascular endothelial growth factor receptor-2; IL, interleukin.

**Table 2:** Clinical studies on amplifying mRNA cancer vaccines.

Vaccine name	Source of RdRp	Antigen	Tumor	Concomitant drug	ClinicalTrials.gov Identifier	Recruitment Status	Phase	Reference
AVX901	VEEV	HER2	HER2+ cancer	/	NCT01526473	Completed	Phase I	(49)
AVX901	VEEV	HER2	HER2+ Breast/breast	Pembrolizumab	NCT03632941	Recruiting	Phase II	(50)
AVX701	VEEV	CEA	Stage III colon cancer	/	NCT01890213	Completed	Phase I	(19)
AVX701	VEEV	CEA	Colorectal cancer Breast cancer Lung cancer Pancreatic cancer Colon cancer	/	NCT00529984	Completed	Phase I/Phase II	(19, 51)
Vvax001	SFV	E6 E7	Cervical	/	NCT03141463	Completed	Phase I	(52, 53)
GRT-902	VEEV	Neoantigen	Colonic neoplasms Colorectal neoplasms	Atezolizumab Ipilimumab	NCT05456165	Terminated	Phase II	(54)
JCXH-211	VEEV	IL-12	Cutaneous tumor Malignant solid tumor	/	NCT05727839	Recruiting	Phase I	(55)

### 4.1 Progress on preclinical research of amplifying mRNA cancer vaccines

At present, amplifying mRNA cancer vaccines are used for the high-level expression of tumor antigens. These vaccines have demonstrated the ability to elicit strong cellular and humoral immune responses in animal models (12). Preclinical studies in this area have primarily focused on tumor-associated antigens (TAAs), tumor-specific antigens (TSAs), and immunomodulatory molecules and have achieved promising therapeutic effects in various mouse tumor models.

Differences in the levels of expression of TAAs between tumors and normal cells serve as possible targets for cancer vaccine therapy. Currently, the design of amplifying mRNA cancer vaccines in the preclinical stage mainly involves the targeting of TAAs, including tyrosinase, pMEL17/gp100, gp75/tyrosinase-related protein (TRP)-1, MART-1/melan-A, and dopachrome tautomase/TRP-2 which are preferentially expressed in melanoma cells. Avogadri et al.’s research suggested that amplifying mRNA cancer vaccine based on VEEV vector expressing TRP-2 induced Humoral immunity against TRP-2, played a role in immunotherapy of Melanoma, and cooperated with tumor specific CD8 T cell reaction (45). Six-transmembrane epithelial antigen of the prostate (STEAP) is highly overexpressed in human prostate cancer tissue. Garcia-Hernandez et al. used VEE virus-like replicon particles (VEE VRPs) for vaccination in prophylactic and therapeutic mouse prostate tumor models, and found that vaccination increased the overall survival rate of prostate tumor-bearing mice (48).

TSAs, also known as neoantigens, originate from random somatic cell mutations within tumor cells. These antigens do not exist in normal cells and are thus ideal targets for cancer vaccines. More than 99% of cervical cancers express the E6 and E7 oncogenes, which are necessary for the malignant phenotype. Daemen et al. used a SFV vector to carry an E6E7 fusion protein that possessed higher stability than the individual E6 and E7 proteins. Compared with mice vaccinated with SFV vectors independently expressing E6 and E7 proteins, mice immunized with the SFV vector expressing the E6E7 fusion protein exhibited stronger cytotoxic T lymphocyte (CTL) responses. Immunization of tumor-bearing mice led to tumor regression and eradication, indicating good oncotherapeutic effects (39).

IL-12 can induce the differentiation of naïve T-cells into T helper type 1 (Th1) cells, which are essential for cellular immunity-mediated antitumor responses. The antitumor effects of IL-12 are exerted through the activation of CTLs and natural killer (NK) cells and the induction of interferon-gamma (IFN-γ) production. Rodriguez Madoz et al. used SFV vector expressing IL-12 to treat MC38 cell line colon cancer mice, which significantly enhanced the anti-tumor immune response of mice, leading to tumor regression and complete eradication (42). IL-18 can increase NK cell viability and T-cell proliferation, promote the secretion of IFN-γ and granulocyte-macrophage colony-stimulating factor (GM-CSF) by NK and T-cells, and enhance the production of Th1-type cytokines, which are associated with antitumor CTL responses. Yamanaka et al. combined intratumoral injection of IL-18 bound by a genetically engineered SFV vector with systemic administration of IL-12 to induce responses from antibrain tumor-specific CD4^+^ and CD8^+^ T-cells and NK cells. This strategy led to a significant enhancement in antitumor effects (41).

Preclinical studies have shown that amplifying mRNA cancer vaccines can evoke strong humoral and cellular immune responses, effectively inhibiting f tumors growth such as melanoma, cervical cancer, and prostate cancer. These results may serve as valuable reference data for subsequent clinical studies.

### 4.2 Progress on clinical research of amplifying mRNA cancer vaccines

Presently, many candidate amplifying mRNA cancer vaccines have entered clinical testing. Various clinical trials have revealed that amplifying mRNA cancer vaccines enhance T-cell immune responses and improve patient outcomes and survival. Therefore, these vaccines provide new tools and support for oncotherapy. On November 9, 2022, JCXH-211 was approved for phase I clinical testing by the National Medical Products Administration (NMPA) of China. This is a novel samRNA-based therapeutic encoding human IL-12 for the treatment of various solid tumors that is presently in the subject recruitment phase (55). Vvax001, a therapeutic cancer vaccine based on a self-amplifying SFV vector, is currently undergoing phase I clinical trial testing for the evaluation of its immunogenicity, safety, and tolerability in the treatment of human papillomavirus (HPV)-induced cancers (52). AVX701 is a self-amplifying VEEV vector-based cancer vaccine expressing a modified carcinoembryonic antigen gene (CEA (6D)). Preliminary results of a phase I/II clinical trial in patients with metastatic colorectal cancer revealed that vaccinated patients exhibited stronger T-cell immune responses and longer survival (19, 51). AVX901, an amplifying mRNA vaccine based on a self-amplifying VEEV vector encoding HER2, has completed phase I clinical trial in the treatment of HER2+ breast cancer, and the phase II clinical trial for its concomitant use with pembrolizumab is currently in the recruitment stage (50). In tumors that carry nonsynonymous DNA mutations, human leukocyte antigens on tumor cell surfaces express peptides containing these mutations as nonself antigens. A subset of these mutated peptides serve as neoantigens that are capable of generating T-cell responses targeting tumor cells. GRANITE is a vaccine based on the self-amplifying VEEV vector GRT-R902 and adenoviral vector GRT-R901 that expresses (54) neoantigens. It is used in combination with checkpoint inhibitors for the treatment of advanced metastatic solid tumors and is currently undergoing phase II/III clinical testing.

Notably, there has been a rapid rise in the number of clinical trials of mRNA cancer vaccines. Although clinical trials of mRNA cancer vaccines are still in the early stages, swift advancements achieved in this field suggest that the prospects of amplifying mRNA cancer vaccines remain promising.

## 5 Optimization strategies for amplifying mRNA cancer vaccines

The length of amplifying mRNAs exceeds that of conventional nonamplifying linear mRNAs by 7,000 bp, leading to disadvantages such as higher tendency for mRNA degradation; increased difficulty in the preparation of *in vitro* transcription (IVT)systems; higher complexity in chemistry, manufacture, and control (CMC) processes; low LNP encapsulation efficiency; and low delivery efficiency. In recent years, amplifying RNA cancer vaccines have achieved a series of breakthroughs in stability, translation efficiency, and delivery efficiency, with advancements made mainly in the following areas (1): Design and optimization of amplifying mRNA carrier backbone sequences to enhance the intensity and stability of mRNA expression while reducing the innate immune responses of the host (2); Selection and combination of tumor antigens (3); Optimization of IVT systems (4); Design of novel delivery systems suitable for amplifying mRNAs.

### 5.1 Optimization of amplifying mRNA vector sequences

Amplifying mRNAs activate intracellular pattern recognition receptors during *in vivo* transcription and synthesis, which can inhibit the overall protein translation process within cells. Inhibition of the protein kinase R (PKR) and IFN pathways significantly improves the translation efficiency of amplifying mRNAs *in vivo* and *in vitro* (56, 57). Notably, amplifying mRNAs, which contain a self-replicase sequence with a length of 7–8 kb, have a much longer length than that of conventional linear mRNAs, increasing the demands on mRNA stability. Blakney et al. developed a split replicon (splitzicon) system that demonstrated the self-amplifying characteristics of replicon RNA. The splitzicon system was also used for encoding multiple antigens, providing a novel approach for the design of multivalent RNA vaccines (58). Beissert et al. developed a bipartite taRNA system that demonstrated high-efficiency levels of protein expression similar to those achieved by amplifying mRNAs (20). In recent years, artificial intelligence (AI) tools have been employed for the optimization of mRNA vaccine sequences, leading to significant increases in mRNA half-life, protein expression levels, and thermal stability of mRNAs (59). Li et al. developed an *in vitro* evolution strategy based on the VEEV replicon system and screened two effective mutant replicons, achieving significant increases in the intensity and duration of luciferase expression within mice compared with that of wild-type replicons (60). Conclusively, the optimization of amplifying mRNA vector sequences for reducing the host innate immune responses can significantly enhance the stability and expression efficiency of these vectors.

### 5.2 Optimization of antigen selection and combinations

Antigen selection is crucial for the development of cancer vaccines. Two important features that cancer vaccines should possess are tumor specificity and the ability to induce high-level and controllable antitumor immune responses. Tumor antigens, including TSAs and TAAs, are antigenic molecules that emerge or are overexpressed during the onset and progression of tumors. TSAs, which include HPV E6, E7, CMV pp65, and neoantigens, are newly formed antigens that are either specific to tumor cells or exist only in certain types of tumor but not in normal cells. By contrast, TAAs are not specific to tumor cells and also exist in normal cells and tissues. However, their expression is significantly increased during carcinogenesis. Examples of TAAs include MUC1, which is abnormally overexpressed in many tumor cells, as well as gp100 and MART1, which are respectively overexpressed and abnormally expressed in most melanoma cancer cells. Immunomodulatory molecules such as IL-7, IL-12, IL-15, GM-CSF, and IFN-a and tumor suppressor genes such as p53 and PTEN have also been applied in the antigen design of cancer vaccines. **Table 3** lists the antigens of cancer vaccines currently used in clinical and preclinical studies.

**Table 3:** Cancer vaccine antigens.

	Stage	Antigens
Tumor-associated antigens (TAAs)	Preclinical	CEA (61), gp100 (62), MART1 (63, 64), total tumor RNA (65), cytokeratin 19 (64), PSMA (66), STEAP (67), TRP-1 (68), gp70 (69), MUC1 (70), TRP-2 (71), GM-2 (72)
	Clinical	CEA (NCT00529984 (51), NCT01890213 (19)), MUC1 (NCT00088660 (73)), TRP-2 (NCT01456104 (74)), PSA(NCT01322490 (75)), STEAP1 (NCT00831467 (76)), TPTE (NCT04526899 (77)), PSMA (NCT00831467 (76)), WT1 (NCT02405338 (78)), NY-ESO-1 (NCT02609984 (53)), AML lysate plus mRNA (NCT00514189), hTERT (NCT00923312 (76), NCT00510133), STn (NCT00003638 (79)), GD3 (NCT00037713 (80)), MAGE-C1/2 (NCT00923312 (76)), MAGE-A3 (NCT04526899 (77)), LAMP (NCT00510133), suppressor of cytokine signaling-1 (NCT02688686), PRAME (NCT02405338), 5T4 (NCT00923312 (76)), tyrosinase (NCT04526899 (77)), CA-125 (NCT00418574 (81)), prostate stem cell antigen (NCT00831467), survivin (NCT01197625, NCT02688686), VEGFR2 (UMIN000002500 (81, 82)), EGFRvIII (NCT01480479 (83))
Tumor-specific antigens (TSAs)	Preclinical	HPV E7 (84), human CMV pp65 (85), HPV E6/E7 (86), poly-neo-epitope (87), neoantigens (88)
	Clinical	CMV pp65-LAMP (NCT02529072), Kirsten rat sarcoma viral oncogene mutated proteins (NCT03948763)
Immunomodulatory molecules	Preclinical	IL-12 (89), IL-15 (90), GM-CSF (91), IFN-a (92), OX40L (93), IL-23 (94), IL-36g (95), CD70 (96), constitutively active TLR4 (97)
	Clinical	IL-12 (NCT04710043, NCT03871348, NCT03946800, NCT04455620), TLR7/8-agonist (NCT03291002, NCT03203005), GM-CSF(NCT03871348), RIG-1-agonist (NCT03291002, NCT03203005), IL-23 (NCT03739931 (98)), IL-7 (NCT04710043), human OX40L (NCT03739931 (98)), IL- 15 (NCT03871348), IFN-a (NCT03871348), IL-36g (NCT03739931 (98))
Tumor suppressor genes	Preclinical	PTEN (99), p53 (100)
	Clinical	p53 (NCT02316457, NCT00978913 (101))

Multiple mutation sites usually exist in the antigenic epitopes of tumor cells, with the suppressive tumor microenvironment also exerting certain negative effects on cancer vaccines. In cancer immunotherapy, combination therapy is commonly adopted for enhancing the effects of cancer vaccines. An effective strategy is the simultaneous expression of multiple antigens. Compared with single-antigen OVA1 mRNA, the dual antigens OVA1 and OVA2 mRNAs caused a 30% increase in the antigen-specific activation of T-cells and a two-fold increase in their proliferation. Cotransfection with a combination of CD40L, CD70, and constitutively active TLR4 encoding mRNA was reported to enhance the human dendritic cell-induced activation of T-cells. Interestingly, the adoption of a combination therapy strategy for the treatment of pancreatic ductal adenocarcinoma (PDAC) not only inhibited the expression of PD-1 but also simultaneously activated IL-2Rβγ and suppressed IL-2α signaling, thereby providing excellent therapeutic effects to patients with PDAC (102). Besides tumor antigen targets, mutation sites were also introduced into the nonstructural proteins of the VEE replicon backbone by Li et al. Compared with wild-type VEE, the saRNA modified with nonstructural proteins led to a significant increase in the levels of expression of the target protein in model animals. In addition, the infiltration of CD8^+^ T-cells was enhanced and tumor growth was delayed (60).

The characteristics of amplifying mRNA vaccines should be considered during the formulation of suitable antigen combination strategies targeted toward different tumor types in order to enhance the therapeutic effects of active immunotherapy on tumors.

### 5.3 Optimization of IVT systems

Compared with conventional mRNAs, amplifying mRNAs are longer and contain more secondary structures. Therefore, the optimization of conventional mRNA IVT synthesis systems is necessary. With the application of the Quality by Design (QbD) concept to IVT system optimization, the effects of critical process parameters on critical quality attributes (CQAs) can be rapidly determined. This will enable the development and continuous production of safe and effective RNA vaccines. Samnuan et al. adopted a design of experiment approach to investigate the various factors affecting the yield of amplifying mRNAs produced by the IVT reaction. Subsequently, the optimum component ratios for IVT were determined and used for the synthesis of amplifying mRNAs with high yield and quality (103). Moderna developed a modified T7 RNA polymerase that reduced dsRNA impurities during transcription reactions. This eased the subsequent purification and enhanced the production efficiency (104). Other researchers have found that a novel psychrophilic phage VSW-3 RNA polymerase is capable of reducing terminal and full-length dsRNA byproducts during IVT, which is beneficial for the production of RNAs for *in vivo* use (105). The development of stable IVT systems with high yield and low byproduct formation remains a key area for future research.

### 5.4 Research of delivery systems

Delivery systems are a key aspect of mRNA vaccines. Initially, the direct delivery of naked saRNA into cells was the simplest strategy used. For example, injection of 50 mg of saRNA carrying the influenza virus nucleoprotein was found to induce specific humoral responses in mice. Subsequently, various delivery methods were developed to enhance the stability and delivery efficiency of mRNAs (16). These methods include electroporation, protamine, LNPs, cationic nanoemulsions, polyethylenimine, polymer-based nanoparticles, exosomes, extracellular vesicles, mesoporous silica, and calcium phosphate (9, 106). LNPs can also function as an adjuvant (107). Miao et al. used LNPs containing cyclic lipid components that stimulated the stimulator of interferon genes pathway for the encapsulation of an OVA mRNA vaccine and observed a significant enhancement in its antitumor effects (108). However, the design of novel delivery systems for amplifying mRNA cancer vaccines still holds many key challenges and opportunities. These include the improvement of stability and delivery efficiency of amplifying mRNAs, delivery targeting, and controllability of the activation of the immune system.

## 6 Quality control of amplifying mRNA vaccines

Regarding mRNA vaccines, which are a novel type of vaccine, quality control is of utmost importance. The assurance of good quality, safety, and efficacy will contribute to sufficient trust and confidence among the public in mRNA vaccines and other innovative products and therapies. The World Health Organization, United States Pharmacopeial Convention, and Center for Drug Evaluation of the NMPA of China have published relevant guidelines and documents, thereby providing regulatory agencies with considerations regarding quality control of mRNA vaccines and methods for the analysis of mRNA quality (109–111). In particular, mRNA sequence and integrity, content and purity, capping efficiency, and encapsulation efficiency are key quality parameters specific to mRNA vaccines that determine both their effectiveness and safety.

Currently, nine COVID-19 mRNA vaccines have been approved or granted emergency use authorizations. However, details such as test items, analysis methods, and acceptance criteria of many types of mRNA vaccines have not yet been publicly disclosed (112). Although COVID-19 mRNA vaccines have demonstrated high safety and efficacy, rare serious adverse events such as myocarditis and hypersensitivity reactions have also been reported, suggesting that the quality control of mRNAs requires further research (60, 113, 114) The application of the QbD concept enables the accurate determination of CQAs, key process parameters, and operational spaces of mRNA vaccines, contributing to the establishment of rational control strategies for the production process, which is of utmost importance for the quality control of mRNA vaccines. As mentioned before, mRNA stability is affected by the length of the mRNA fragment. Previous studies have found that the length of mRNA in yeast is negatively correlated with its half-life (115). Amplifying mRNAs carry an additional coding sequence for replicase, which enables their amplification. Consequently, the length of amplifying mRNAs exceeds that of conventional linear mRNAs by 7,000 bp, resulting in poorer stability. Therefore, to achieve the quality control of amplifying mRNA vaccines, further research should be performed on key CQAs such as their stability and *in vivo* replicase activity. In view of the prolonged expression time of amplifying mRNAs, the duration of antigen expression should be calculated for the evaluation of their *in vitro* and *in vivo* activities. Regulatory agencies and vaccine manufacturers should also consider establishing reference standards for the measurement of nucleic acid content, purity, and biological activity of amplifying mRNAs for use in the quality control testing of mRNA vaccines.

## 7 Outlook

Amplifying mRNA cancer vaccines are a highly promising technological platform in the field of cancer treatment. A large number of biopharmaceutical companies and research teams worldwide have made considerable efforts to the research and development of such vaccines, achieving significant progress. To date, five types of amplifying mRNA cancer vaccines have entered clinical testing. However, mRNA vaccines face obstacles such as higher tendency for mRNA degradation, antigen selection and optimization and targeted delivery to tumor. And many issues regarding the research and development, production, quality control, and safety remain to be resolved for amplifying mRNA cancer vaccines. Recently, researchers have proposed the concept of Quality by Digital Design concept as an extension of the QbD approach. Efforts should be made to accelerate the integration of such a concept into the research and production of amplifying mRNA vaccines (116).

Currently, RdRp used for constructing amplifying mRNA cancer vaccines mainly originates from alphaviruses and have a gene sequence length of up to 7 kb. The use of longer gene sequences hinders the production and quality control of mRNA vaccines and may lead to their poorer stability. Therefore, research should be focused on the exploration of RdRp with a shorter gene sequence or the design and optimization of various component elements to reduce the mRNA length in vaccines. Delivery systems are of critical importance for mRNA vaccines. Given the relatively long nucleotide sequences of amplifying mRNA vaccines, existing LNPs may not be suitable for encapsulating longer mRNAs. The optimization of currently available LNPs or development of new delivery systems suited to the encapsulation of longer mRNAs is therefore required. Targeted therapy not only enables precise killing of tumors but also reduces the side-effects of medications. Therefore, the exploration and development of delivery systems with higher targeting capabilities for use with amplifying mRNA cancer vaccines is highly necessary. Many studies have indicated that tumor tissues are generally in a state of hypoxia. Recent studies have also demonstrated that hypoxia within cells or tissues can lead to reduced transfection and expression efficiency of LNP-mRNA. Therefore, further design and optimization of amplifying mRNA cancer vaccines should be performed to enhance their transfection and expression efficiencies within the body.

The relatively long sequences of amplifying mRNA vaccines may also affect the efficiency of IVT. Therefore, further optimization of the reaction conditions and systems of IVT and the development of more efficient IVT enzymes should be carried out. The use of cap analogs from different sources and optimization of the cap analog-guanosine ratio may enhance IVT efficiency. In addition, longer mRNA sequences may also cause premature termination of IVT, resulting in decreased integrity of the generated mRNA sequences. Therefore, optimization of the IVT reaction is required to achieve the generation of complete target sequences. The production of mRNA vaccines also requires a purification step. Considering that the IVT products of amplifying mRNA vaccines may contain a considerable proportion of incomplete mRNAs, it is necessary to develop and optimize suitable purification processes to enhance purification efficiency.

The establishment and validation of quality control methods for amplifying mRNA vaccines should be performed based on specific requirements stipulated in the General Chapter <1220> “The Analytical Procedure Lifecycle” recently released by the USP and the ICH Q2 “Validation of Analytical Procedures” and Q14 “Analytical Procedure Development” draft guidelines published by the International Council for Harmonization of Technical Requirements for Pharmaceuticals for Human Use (ICH) for which public comment is currently being sought (117, 118). This includes the establishment of an analytical target profile, risk analysis and identification, confirmation of CQAs, experimental design, process control, and in-use monitoring. The most prominent characteristic of amplifying mRNA vaccines is their ability to replicate within cells. Therefore, it is important to focus on the quality control of their replicating properties. Given the lack of research on relevant quality control methods, there is a need to develop pertinent methods and establish standards to facilitate the research and development of such vaccines. The relatively long sequences of amplifying mRNAs also cause lower stability of the resultant vaccines. Therefore, it is also crucial to focus on quality control of the markers of integrity of vaccine sequences.

In terms of safety, given that amplifying mRNA vaccines can continuously generate new mRNAs within the body, attention should be paid to the possibility of evoking strong systemic or local inflammatory reactions. The fact that such vaccines encode RdRp signifies that the body will express RdRp after immunization. However, in the case of viral infection in the body post-immunization, RdRp may bind to the RdRp binding site in the viral genome, raising concerns about the promotion of viral genome replication. Further investigation is also needed to determine whether this system might cause the replication of the host mRNA, thereby affecting gene expression in the host.

## Author contributions

CH: Writing – original draft, Writing – review & editing. JL: Writing – original draft, Writing – review & editing. FC: Writing – original draft, Writing – review & editing. YB: Writing – review & editing. QM: Conceptualization, Funding acquisition, Writing – review & editing. MX: Conceptualization, Funding acquisition, Writing – review & editing. ZL: Conceptualization, Funding acquisition, Writing – review & editing.
